# Wide QRS monomorphic tachycardia induced by ajmaline infusion

**DOI:** 10.1016/j.hrcr.2025.02.010

**Published:** 2025-02-15

**Authors:** Pierre-Léo Laporte, Alessandra Pia Porretta, Morgat Charles, Vincent Algalarrondo, Fabrice Extramiana

**Affiliations:** Reference Center for Inherited Arrhythmic Syndromes, Hôpital Bichat, Assistance Publique–Hôpitaux de Paris, Université Paris Cité, Paris, France

**Keywords:** Sodium channel blocker test, Ventricular tachycardia, Bundle branch reentrant tachycardia, Brugada syndrome, Drug-induced Brugada syndrome

**Key Teaching Points**

• Sodium channel blocker test is not a risk-free test.
• Tachycardias induced during diagnostic ajmaline infusion can be bundle-to-bundle tachycardia.
• Tachycardias with QRS complexes identical to the baseline morphology can be ventricular tachycardia.
• A baseline prolonged HV interval necessitates increased caution when administering ajmaline, as it can further prolong conduction and elevate the risk of arrhythmias.

## Introduction

The sodium channel blocker test is a useful tool for diagnosing Brugada syndrome in the absence of a spontaneous type 1 pattern. However, it is not a risk-free test. Indeed, in 0.5%–1% of cases, a malignant ventricular arrhythmia may occur.^1^ We present the case of a patient who experienced an atypical ventricular arrhythmia during a sodium channel blocker test.

## Case report

**Figure 1:**
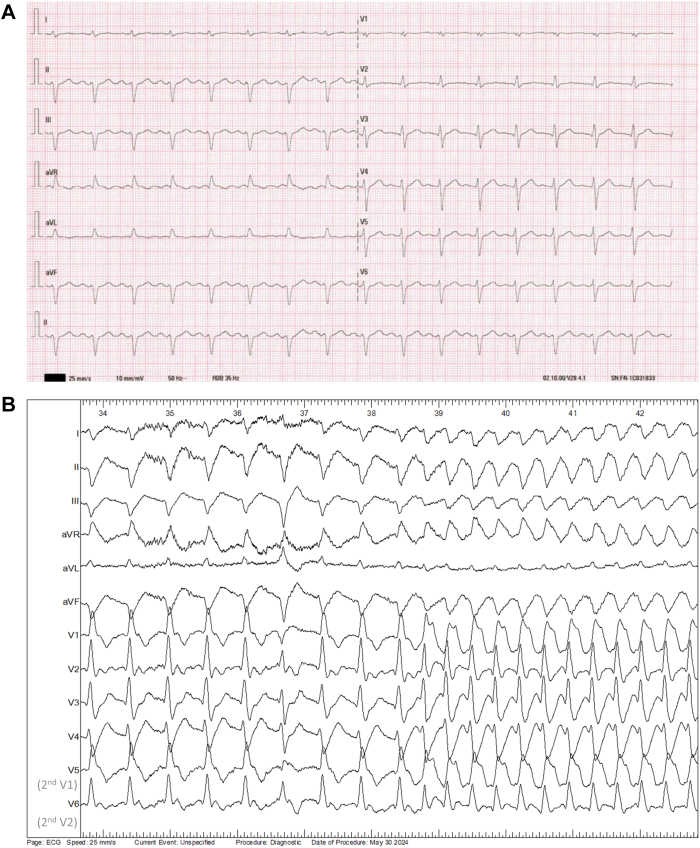

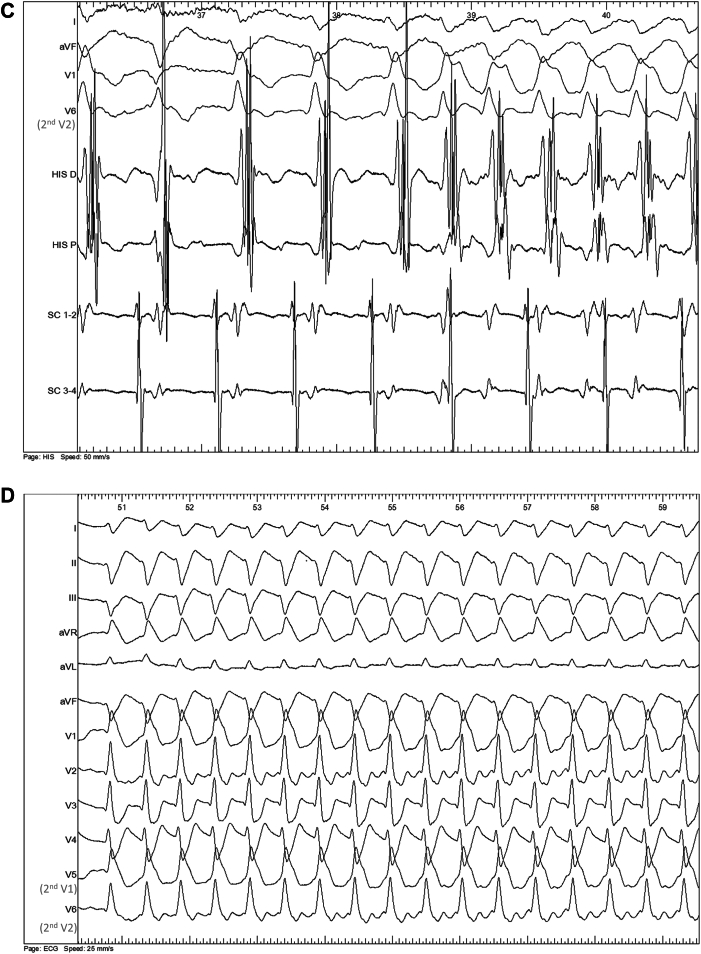
**A:** Baseline electrocardiogram (ECG). **B:** 4th minute ajmaline infusion ECG initiation of tachycardia at 110 beats/min without changes in QRS morphology (widened QRS); 25 mm/s, 10 mm/mV. V5 and V6 electrodes are positioned on second intercostal space. **C:** Endocavitary recording at the 4th minute of ajmaline infusion. Four lead ECG recorded (D1, AVF, V1, V6; V6 electrode is positioned on second intercostal space), 2 intracardiac quadripolar catheters (His D/P in the ventricle and SC 1–2/3–4 in the coronary sinus). Initiation of tachycardia with atrioventricular dissociation. **D:** 4th minute ajmaline infusion ECG. Diagnostic of Brugada syndrome (type 1 pattern); 25 mm/s, 10 mm/mV V5 and V6 electrodes are positioned on second intercostal space. D/P = distal/proximal; SC = coronary sinus.

A 57-year-old patient with no medical and familial history (no sudden cardiac death in first- or second-degree relatives) was admitted to the cardiac intensive care unit after resuscitation from a cardiorespiratory arrest due to ventricular fibrillation. Etiologic workup included a negative toxicology screening and an electrocardiogram showing sinus rhythm with incomplete right bundle branch block and left axis deviation (Figure 1A). Transthoracic echocardiography revealed a left ventricular ejection fraction of 60%, with no segmental wall motion abnormalities of the left or right ventricles. Coronary angiography, including intracoronary Methergin injection,^2^ cardiac magnetic resonance imaging, and cranio-thoraco-abdominopelvic computed tomography scan were normal.

According to guidelines, we also performed an electrophysiological study, which showed a delayed HV interval (72 ms), a normal atrioventricular node effective refractory period (240 ms), no accessory pathway, and a negative programmed ventricular stimulation (2 sites, 2 extrastimuli [600 and 400 ms] up to 4 [minimum coupling 200 ms], with and without isoproterenol). Pharmacologic tests^2^ included an infusion of sodium triphosadenine causing blocked P waves without unmasking accessory pathway and epinephrine infusion^3^ up to stage 5 without pathologic QT interval prolongation or ventricular ectopy. Ajmaline infusion (1 mg/kg in 5 minutes intravenous infusion)^4^ revealed a type 1 Brugada pattern, at the 4th minute after the beginning of the infusion, with progressive QRS widening and spontaneous onset of regular wide QRS monomorphic tachycardia with a cycle length of 550 ms (Figure 1B–1D). QRS morphology during tachycardia was almost identical to that observed in the sinus rhythm preceding the onset of tachycardia (Figure 1B). However, the intracardiac recording showed atrioventricular dissociation (Figure 1C). The ventricular tachycardia (VT) resolved within 2 minutes after administration of 8.4% molar bicarbonates, without endocavitary maneuvers.

In terms of follow-up, the patient underwent genotyping to look for a sequence variation in the SCN5A gene, but the results are not yet available. After the electrophysiological study and, given the high risk of recurrent cardiopulmonary arrest,^5^^,^^6^ we decided to implant an implantable cardioverter-defibrillator. To date, the patient has had no recurrence of sustained ventricular arrhythmias.

## Discussion

Since 2013, the pharmacologic challenge test has been recommended for diagnostic purposes for Brugada syndrome in the absence of a spontaneous type 1 electrocardiogram pattern.^7^ The test is considered positive when a type 1 electrocardiogram pattern appears during the infusion of ajmaline (1 mg/kg over 5 minutes). The proarrhythmic effects of ajmaline when dealing with patients with Brugada are well-known, but are most often responsible for frequent premature ventricular contractions, nonsustained or sustained polymorphic VT or ventricular fibrillation, with reported ventricular fibrillation rates ranging from 0.3% to 10% (mean 1.3%).^8^, ^9^, ^10^, ^11^, ^12^, ^13^ There have been anecdotal reports of refractory ventricular fibrillation occurring during ajmaline infusion, requiring extended resuscitation and/or the use of venoarterial extracorporeal membrane oxygenation.^10^^,^^14^

Rare cases of monomorphic VT have also been reported.^12^^,^^15^ These tachycardias initially presented as nonsustained pleomorphic VTs, which then evolved into monomorphic VTs with a right bundle branch block pattern. QRS widening is an expected effect of ajmaline related to sodium channel blockade that impairs action potential propagation within the conduction system.^13^ In the presence of a pre-existing conduction disturbance in 1 branch, a unidirectional block can occur, leading to bundle branch reentrant tachycardia. A spontaneous case of bundle branch reentrant VT has also been reported in a patient with Brugada,^16^ independently of any ajmaline infusion, which may suggest a link between arrhythmias and conduction disturbances observed in patients with Brugada.

In our case, the diagnosis of VT is definitive (as evidenced by the atrioventricular dissociation visible in Figure 1C), despite a QRS morphology almost identical to that of the sinus rhythm, and the most plausible tachycardia mechanism seems to be bundle-branch reentry. Another possible diagnosis could have been septal VT, potentially using parts of the conduction pathways. However, this diagnosis was excluded due to the absence of echocardiographic scar, pathologic late gadolinium enhancement on cardiac magnetic resonance imaging, and a negative programmed ventricular stimulation. Unfortunately, we did not have a catheter positioned on the His bundle or right bundle branch to demonstrate that the His potential preceded the right bundle potential and the surface QRS, or that the HV interval was longer than in sinus rhythm. Similarly, we did not perform atrial entrainment maneuvers or demonstrate that variations in HH intervals preceded variations in VV intervals.

It is important to emphasize that safety protocols and management strategies for ventricular arrhythmias during pharmacologic testing are well-established. These protocols have been published in guidelines^17^ and are summarized as follows: criteria for stopping the infusion of the pharmacologic agent and antagonizing it with an 8.4% molar bicarbonate solution include QRS widening by ≥130% above baseline and the occurrence of premature ventricular contractions, doublets, or triplets.

## Conclusion

This report is the first to suggest that tachycardias that potentially occur during ajmaline infusion for diagnosing Brugada syndrome may be bundle branch reentrant VTs. The pharmacologic testing with ajmaline in patients with Brugada requires particular caution due to potential ajmaline pro-arrhythmogenic effects and the presence of a baseline prolonged HV interval warrants heightened caution, as it may exacerbate conduction delays. In addition, tachycardias that present with QRS complexes identical to the baseline morphology always deserve careful evaluation, as in some cases, these may represent VT. Therefore, pharmacologic induction tests should be performed under the defined and necessary safety conditions, adhering to the established guidelines.^1^^,^^17^^,^^18^

## Disclosures

The authors have no conflicts of interest to disclose.
